# Use of Human Pluripotent Stem Cells to Define Initiating Molecular Mechanisms of Cataract for Anti-Cataract Drug Discovery

**DOI:** 10.3390/cells8101269

**Published:** 2019-10-17

**Authors:** Chitra Umala Dewi, Michael D. O’Connor

**Affiliations:** School of Medicine, Western Sydney University, Campbelltown, NSW 2560, Australia; 18891209@student.westernsydney.edu.au

**Keywords:** human pluripotent stem cell, lens, micro-lens, cataract, bioinformatics, risk factor, regeneration, ROR1 cells, anti-cataract drug

## Abstract

Cataract is a leading cause of blindness worldwide. Currently, restoration of vision in cataract patients requires surgical removal of the cataract. Due to the large and increasing number of cataract patients, the annual cost of surgical cataract treatment amounts to billions of dollars. Limited access to functional human lens tissue during the early stages of cataract formation has hampered efforts to develop effective anti-cataract drugs. The ability of human pluripotent stem (PS) cells to make large numbers of normal or diseased human cell types raises the possibility that human PS cells may provide a new avenue for defining the molecular mechanisms responsible for different types of human cataract. Towards this end, methods have been established to differentiate human PS cells into both lens cells and transparent, light-focusing human micro-lenses. Sensitive and quantitative assays to measure light transmittance and focusing ability of human PS cell-derived micro-lenses have also been developed. This review will, therefore, examine how human PS cell-derived lens cells and micro-lenses might provide a new avenue for development of much-needed drugs to treat human cataract.

## 1. Introduction

Cataract is a condition in which light transmission through the ocular lens is decreased, resulting in reduced vision and blindness. The ability to define the initiating molecular mechanisms of human cataract formation—and, therefore, effective treatments to inhibit or delay cataract progression—has largely been hampered by the lack of access to functional human lens tissue at the initial stages of cataract formation. The ability of human pluripotent stem (PS) cells to (i) self-renew and (ii) differentiate into any cell type of the body, means human PS cells can provide a large-scale source of normal or diseased human cells for research [1,2,3,4]. Consequently, human PS cells are enabling new research approaches into human cell and tissue development, elucidation of molecular disease mechanisms, drug discovery and toxicity assessments, and investigation of candidate cell-based therapies. This review will explore how human PS cell technology is being applied to cataract research, with particular emphasis on cataract disease modelling, drug discovery and toxicity assessment.

## 2. Human PS Cell-Derived Organoids

The types of human PS cells most widely used for research are embryonic stem cells [5,6] and induced pluripotent stem cells [7,8,9]. Cell culture maintenance of human PS cells involves non-trivial tasks compared to culture of non-pluripotent cell lines. This is due to human PS cells being highly sensitive to variations in basic culture parameters, including the size of cell aggregates, cell and/or cell-aggregate density, time in culture, growth factor and extracellular matrix composition and concentrations, etc.

Significant efforts were made worldwide to identify effective proliferation and maintenance conditions for human PS cells. A comparison of published culture media by the International Stem Cell Initiative identified three media conditions capable of sustained maintenance of multiple human PS cell lines across five independent laboratories [10]. Nowadays, commercially available human PS cell media provide defined, feeder-free culture conditions for robust and reproducible expansion of human PS cells.

As a consequence of having reliable human PS cell maintenance conditions, human PS cell differentiation strategies are now being improved to the extent where generating large numbers of purified, differentiated cells is possible for a variety of cell types. Moreover, human PS cell differentiation strategies have begun to evolve to the point where they can reproducibly generate large numbers of small, three-dimensional human tissues, termed ‘organoids’. These stem-cell-derived organoids mimic aspects of the cellular arrangement, and to varying extents, the overall function, of human tissues [11,12,13]. Human PS cell-derived organoids, therefore, have the potential to provide new and powerful tools for elucidating molecular mechanisms of disease progression that are specific to individual disease risk factors, as well as associated drug discovery studies [14,15,16].

## 3. Human PS Cell-Derived Lens Epithelial Cells and Micro-Lenses

As summarized by Murphy et al., a number of methods have been used to produce lens epithelial cells (LECs) at different levels of purity from human pluripotent stem cells [17]. The method that generates the most purified LEC population involves cell purification via an antibody that detects the ROR1 (receptor tyrosine kinase-like orphan receptor 1) cell surface antigen. Subsequent aggregation and culture of these purified LECs generates micro-lenses that share key properties of primary human lenses, including:

(i) The ability to transmit and focus light;
(ii) A cellular architecture consisting of LECs and a mass of lens fibre cells;
(iii) Expression and accumulation of lens-specific crystallin proteins;
(iv) Ultrastructural changes, including lens fibre cell denucleation and formation of complex membrane interdigitations.

Of the various methods used to produce lens cells from human PS cells, the ROR1-LEC/micro-lens system shares the largest number of functional lens properties with primary human lenses, in particular, the ability to focus light. Accordingly, this review will focus on how the ROR1-LEC/micro-lens system might be used to investigate cataract formation.

To test whether human PS cell-derived micro-lenses might be suitable for investigating cataract formation in vitro, ROR1-expressing LECs were exposed to a drug (Vx-770) suspected of causing non-congenital cataract in young cystic fibrosis patients [18,19]. Strikingly, micro-lenses treated with high Vx-770 concentrations lost their ability to transmit and focus light [17]. These findings suggest human PS cell-derived, ROR1-expressing LECs and micro-lenses could aid identification of the specific, initiating cataract molecular mechanisms that result from different cataract risk factors. The ability to precisely alter the environment in which stem-cell-derived, human lens cells and micro-lenses are cultured—for example, by changing the concentration of oxygen (hypoxia, normoxia, hyperoxia), nutrients, drugs, etc.—provides a new opportunity to define how individual or combinations of factors lead to cataract initiation and progression.

## 4. Cataract: Impairment of Lens Function

**Figure 1:**
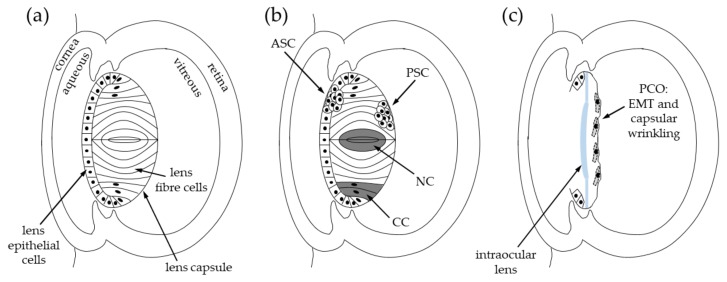
Diagram of the lens and cataract types. (**a**) Location of the lens, lens epithelial cells (LECs) and lens fibre cells within the eye. Black dots indicate nuclei within epithelial cells and differentiating fibre cells. (**b**) Location of different types of cataract within the lens, including anterior subcapsular cataract (ASC), posterior subcapsular cataract (PSC), cortical cataract (CC) and nuclear cataract (NC). (**c**) Location of posterior capsule opacification (PCO) in the lens capsular bag after cataract surgery. Lens epithelial cells undergo an epithelial-to-mesenchymal transition (EMT) and cause capsular wrinkling.

The term cataract describes an opacification of all or specific regions of the ocular lens. Cataracts can cause reduced vision and blindness by impairing the lens’ ability to focus light onto the retina. Various anatomical and molecular changes have been associated with cataractous lenses including brunescence, formation of light scattering particles, and localized changes in the refractive index [20,21,22]. These effects can reduce the luminosity, contrast and/or clarity (‘acuity’) of images being received by the retina. The magnitude of these effects depends on the cataract morphology and the proportion of the pupillary area occupied by the cataract (see Figure 1).

Cataracts typically occur in adults, though they can also occur in children—for example, congenital and traumatic cataracts. In adults, cataracts most often present slowly and painlessly. Due to the subtle progression of cataract formation, many patients are often unaware of the initial changes in their vision [23]. The gradual nature of cataract formation, together with the inability to access lens tissue at the early stages of disease initiation, has made identification of risk-factor-specific mechanisms of cataract formation highly challenging.

## 5. Types of Cataract

Cataracts can be defined by different characteristics, for example, their location within the lens.

- Nuclear cataract is located in the center (or ‘nucleus’) of the lens. With ageing, the lens nucleus can darken, changing from clear to yellow and even brown; a process called brunescence [24,25].
- Cortical cataract forms within the peripheral layers of lens fibre cells, situated outside of the lens nucleus. Cortical cataract (e.g., diabetic cataract) often has a wedge- or spoke-like appearance pointing towards the centre of the lens, and is frequently associated with glare [25,26].
- Anterior subcapsular cataract arises within the anterior LEC monolayer; it results from abnormal growth and/or differentiation of lens epithelial cells, resulting in fibrotic plaques [25,27].
- Posterior subcapsular cataract forms under the posterior lens capsule due to abnormal growth and differentiation of LECs or immature lens fibre cells [28]; it can cause light sensitivity and glare [25,29].
- Posterior capsule opacification (PCO) is the most common complication of primary cataract surgery. PCO develops from residual LECs not removed during primary cataract surgery. These cells proliferate, migrate and undergo abnormal differentiation on the posterior capsule, causing capsular wrinkling [30].

## 6. Cataract Surgery

Currently, cataract development cannot be delayed. However, cataracts can be surgically treated via phacoemulsification to remove the lens cells and associated cataract—this typically happens after significant loss of vision has occurred. Lens cell removal is followed by implantation of a rigid plastic intraocular lens that restores a fixed focal point onto the retina (Figure 1). Cataract surgery generally restores vision immediately, and so has become common place in developed countries where the required equipment and expertise are readily available. Vision restoration in cataract patients restores more than just the lifestyle and functioning capacity of the patient. Due to the care that cataract patients require as a result of their vision loss, vision restoration via cataract surgery often has wider effects on family members and caregivers—for instance, freeing time for family members to return to the workforce [31,32].

Millions of cataract surgeries are performed worldwide each year, costing billions of dollars. In the USA, for 2004, the direct medical costs attributed to cataract were estimated at $6.8 billion [33]. Cataracts also contribute to an estimated $8 billion in annual productivity losses per year in the USA [33]. Additionally, estimates suggest between $65 and $157 million is spent annually treating PCO [34,35]. Despite this large investment in cataract health services, cataract continues to be a leading cause of blindness worldwide, with the number of patients with low vision or blindness due to cataract having increased from ~50 million in 1990 to ~65 million in 2015 [36]. Additional, less frequent but large-impact complications of cataract surgery include: refractive error, retinal detachment and visual impairment [37,38]. As a result of these side-effects, there is significant patient, clinical, industry and academic interest in identifying effective anti-cataract drugs to delay cataract formation. Notably, it has been estimated that delaying cataract formation by 10 years could almost halve the number of cataract surgeries required [39]. At present, however, no effective anti-cataract drug has been identified for humans. This is largely due to the inability to access human lens material during the early stages of cataract formation.

## 7. Cataract Risk Factors

Cataracts are often categorized based on the location in which they develop within a lens, though it is unlikely the molecular pathology of cataract formation is the same for all cataract subtypes. While some later aspects of cataract formation may be common to more than one type of cataract—for example, light-scattering particles such as protein aggregates [40] or multi-lamellar bodies [41]—it is likely that at least some of the initiating mechanisms of cataract formation are unique to each particular cataract risk factor responsible for cataract formation.

Various environmental cataract risk factors have been identified, including age [42], heat [43,44], UV light [45,46,47], smoking [48], diabetes [49], oxidation [24] and some drugs, such as glucocorticoids [28]. Over 300 genetic mutations have also been associated with congenital cataract or adult cataract [50], including crystallin [51,52] and connexin mutations [53]. Partial molecular mechanisms have been postulated for some forms of cataract, such as congenital cataracts caused by connexin mutations [54] and age-related cataract [55,56].

Much of our knowledge of lens biology has come from in vitro and in vivo studies of animal lens and cataract development, including drosophila, mice, rats, dogs, cows, salamanders, rabbits, and kangaroo [24,57,58,59,60,61,62,63,64,65,66,67,68,69]. These animal-based studies have been valuable in providing a framework for understanding human lens and cataract biology. Nevertheless, animal models are poorly predictive of human biology [70,71,72]. While various animal models have been used to test the ability of different molecules to delay cataract formation [73,74], to date, no effective anti-cataract drug has been identified for human patients.

From a practical perspective, in vitro models for anti-cataract drug discovery benefit from the ability to be performed at a small scale (for targeted studies of drug candidates) to large scale (for drug screening assays). These requirements mean that whole animal lenses are poorly suited to many anti-cataract drug discovery approaches. In addition to scalability issues and inherent species-specific differences compared to humans, in vitro animal-based cataract models—such as explanted lens tissue—often lack the three-dimensional arrangement of normal lens tissue. As a result, cataract models that rely on explanted lens tissue [75,76] may not mimic some important lens parameters that occur in vivo, such as lens capsule-mediated access of drugs to lens cells.

To try and avoid the limitations inherent to animal-based investigations of cataract formation [56], primary human lens material has been used [77]. This includes the human lens capsular bag model, emulsified lens material obtained through cataract surgery, and small numbers of donated human lenses. These studies have defined important differences between late-stage adult cataract and aged, non-cataractous lenses. This includes a role for the lens epithelium in maintaining lens health through ion transport/homeostasis, and subsequent circulation of anti-oxidants through the lens [55] that appears to be affected in late-stage cataract. Nevertheless, key aspects of lens circulation still need to be defined, including how cataract progression is affected by functional heterogeneity within the lens epithelium—and more research is needed. However, the small amount of primary human material that can be accessed—together with the late stage of cataractogenesis obtained and the irregular supply of primary human material—makes it challenging to use these models for defining initiating mechanisms of primary cataract formation. Immortalized human cell lines have been employed as an alternative [78,79], but how closely these cells reflect normal lens biology is debatable. Furthermore, human lens cell lines have not been shown to develop into transparent, light-focusing three-dimensional lens tissue—a key feature of normal lens biology.

## 8. Defining Cataract Mechanisms with ROR1-Expressing Lens Cells and Micro-Lenses

As described in Section 3, human PS cells can be used to generate large numbers of ROR1-expressing LECs and micro-lenses. Exposing these micro-lenses to clinically relevant doses of a potential cataract risk factor (Vx-770) reduced micro-lens transparency and focusing [17]. The ability to collect cell culture samples at any time after treatment suggests ROR1-expressing LECs and micro-lenses could provide valuable new systems for defining the initiating mechanisms of PCO and primary human cataract. Being able to control which cataract risk factor or combinations of risk factors the lens cells and micro-lenses are exposed to could provide new insights into the initial stages of cataract formation—insights that cannot be obtained using mouse lenses or primary human lens tissue. For example, an interesting approach could be to study human PS-cell-derived micro-lenses that possess crystallin mutations, with and without exposure to environmental cataract risk factors. Defining the molecular consequences of cataract risk factors on functional human micro-lenses could potentially also provide new information on lens-protection mechanisms. These new insights could then lead to new candidate anti-cataract drug targets and/or anti-cataract drugs.

**Figure 2:**
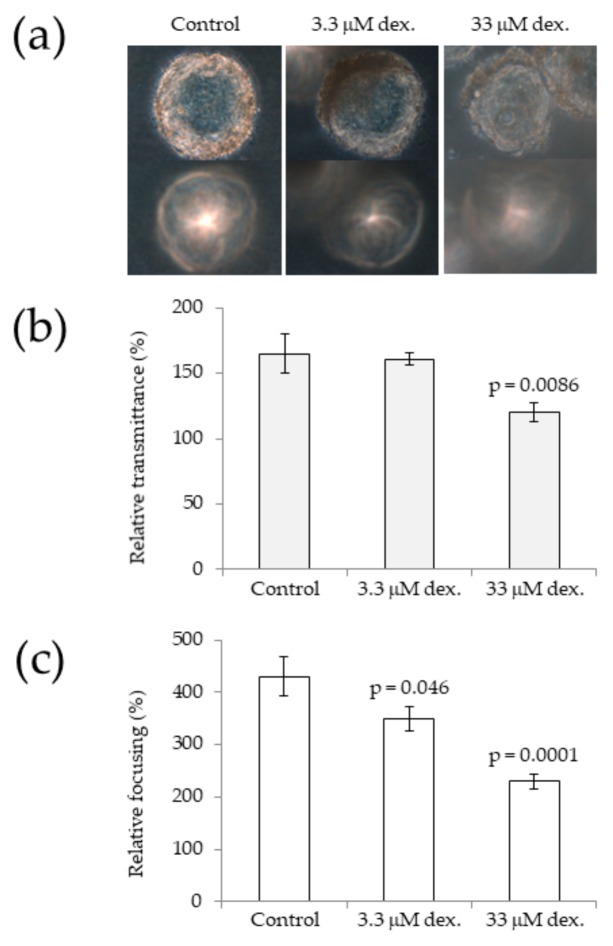
Dexamethasone induces cataract-like effects in human micro-lenses after 8 days of treatment. (**a**) Phase contrast images show that exposing micro-lenses to increasing concentrations of dexamethasone (dex.) decreases light transmittance (top row) and focusing ability (bottom row). (**b**) Quantitative image analysis showing that increasing dexamethasone concentration significantly decreases micro-lens light transmittance compared to control (vehicle-only) treatment. (**c**) Quantitative image analysis showing that increasing dexamethasone treatment decreases micro-lens focusing ability compared to control (vehicle-only) treatment. The micro-lenses were derived from human-induced pluripotent stem cells, and were cultured for 10 days until light focusing occurred, after which time, treatment was initiated. Error bars represent standard error of the mean; eight micro-lenses from three independent experiments were analysed for each treatment.

The variety of cataract risk factors available for modelling via stem-cell-derived LECs and micro-lenses (noted above) can be prioritized based on the complexity required to replicate particular risk factors in vitro. Currently, used drugs that have primary cataract as a side-effect might be the simplest cataract risk factors to investigate, due to the ease in which they can be added to culture media over a range of clinically relevant concentrations. For example, dexamethasone is routinely prescribed to treat a variety of disorders, such as rheumatoid arthritis [80], ocular inflammation [81], and post-surgical inflammation [82,83]. Notably, long-term use of dexamethasone has been associated with posterior subcapsular cataract [84,85,86]. Preliminary data from our group has shown that micro-lenses exposed to dexamethasone have reduced light transmission and focusing ability (Figure 2). These data suggest that further investigation of this system could identify how dexamethasone-induced cataract occurs in humans.

## 9. Drug Toxicity Assessment Using ROR1-Expressing Lens Cells and Micro-Lenses

In addition to their potential for defining initiating mechanisms of human cataract formation, ROR1-expressing LECs and micro-lenses have significant potential for providing a new, large-scale and high-throughput system for assessing lens toxicity assessment. For example, new drugs could be tested using the micro-lenses to quantify their effects on human micro-lens transparency and/or focusing. Such an application would be consistent with how human PS-cell-derived cardiomyocytes have been approved by the US Food and Drug Administration for cardiotoxicity assessment of new drugs [87]. Alternatively, older drugs that failed pre-clinical development due to the appearance of cataracts in animal models could be re-investigated using the micro-lens system—in order to determine whether they similarly cause cataract in functional human lens tissue.

Defining drug-induced molecular consequences within human micro-lenses could also identify potential cataract biomarkers to stratify patients at low- vs. high-risk of cataract formation. For example, higher concentrations of particular drugs may cause cataract formation [17]. Therefore, identifying patients that experience higher ocular drug concentrations (e.g., by quantifying drug concentration in the tear film) may enable stratification of patients into low- vs. high-risk of cataract formation. In turn, this information could identify patients in need of more frequent assessment of lens/eye health, and/or enable improved drug prescribing to minimize cataract formation in patients.

Defining molecular mechanisms of cataract formation using micro-lenses could also potentially identify candidate co-therapies to avoid cataract formation—in a similar way to co-therapies being used to avoid side-effects of other drugs. For example, folate is co-prescribed with methotrexate for rheumatoid arthritis/rheumatic diseases, in order to avoid hepatotoxicity and gastrointestinal side-effects caused by methotrexate-based treatment [88]. Defining the molecular mechanisms of drug-induced cataract formation could offer similar opportunities for co-therapy development to avoid cataract formation.

## 10. Conclusions and Future Perspectives

Human PS-cell-derived lens cells and micro-lenses can provide a large-scale source of functional human lens tissue, possessing a range of morphological, molecular and functional similarities to primary human lenses. These ROR1-expressing lens cells and micro-lenses can be used to model individual or combined cataract risk factors, and associated human cataract initiation events in vitro. They can also be applied to small-scale or large-scale drug discovery and toxicity assays. Given the large annual financial burden cataract surgery places on health systems worldwide, investigating human cataract formation using ROR1-expressing lens cells and micro-lenses has significant potential to reduce the personal, social and economic consequences of cataract.

## References

[1] Silva J., Smith A. (2008). Capturing Pluripotency. Cell.

[2] Liras A. (2010). Future research and therapeutic applications of human stem cells: General, regulatory, and bioethical aspects. J. Transl. Med..

[3] Fernandez T.D.S., Fernandez C.D.S., Mencalha A.L. (2013). Human Induced Pluripotent Stem Cells from Basic Research to Potential Clinical Applications in Cancer. BioMed Res. Int..

[4] Pomp O., Colman A. (2013). Disease modelling using induced pluripotent stem cells: status and prospects. Bioessays.

[5] Thomson J.A., Itskovitz-Eldor J., Shapiro S.S., Waknitz M.A., Swiergiel J.J., Marshall V.S., Jones J.M. (1998). Embryonic stem cell lines derived from human blastocysts. Science.

[6] Reubinoff B.E., Pera M.F., Fong C.-Y., Trounson A., Bongso A. (2000). Embryonic stem cell lines from human blastocysts: somatic differentiation in vitro. Nat. Biotechnol..

[7] Yu J., Vodyanik M.A., Smuga-Otto K., Antosiewicz-Bourget J., Frane J.L., Tian S., Nie J., Jonsdottir G.A., Ruotti V., Stewart R. (2007). Induced Pluripotent Stem Cell Lines Derived from Human Somatic Cells. Science.

[8] Takahashi K., Tanabe K., Ohnuki M., Narita M., Ichisaka T., Tomoda K., Yamanaka S. (2007). Induction of pluripotent stem cells from adult human fibroblasts by defined factors. Cell.

[9] Park I.H., Zhao R., West J.A., Yabuuchi A., Huo H., Ince T.A., Lerou P.H., Lensch M.W., Daley G.Q. (2008). Reprogramming of human somatic cells to pluripotency with defined factors. Nature.

[10] Akopian V., Andrews P.W., Beil S., Benvenisty N., Brehm J., Christie M., Ford A., Fox V., Gokhale P.J., Healy L. (2010). Comparison of defined culture systems for feeder cell free propagation of human embryonic stem cells. In Vitro Cell. Dev. Biol. Anim..

[11] Clevers H. (2016). Modeling Development and Disease with Organoids. Cell.

[12] Kretzschmar K., Clevers H. (2016). Organoids: Modeling Development and the Stem Cell Niche in a Dish. Dev. Cell.

[13] Ho B., Pek N., Soh B.-S. (2018). Disease Modeling Using 3D Organoids Derived from Human Induced Pluripotent Stem Cells. Int. J. Mol. Sci..

[14] Avior Y., Sagi I., Benvenisty N. (2016). Pluripotent stem cells in disease modelling and drug discovery. Nat. Rev. Mol. Cell Boil..

[15] Rowe R.G., Daley G.Q. (2019). Induced pluripotent stem cells in disease modelling and drug discovery. Nat. Rev. Genet..

[16] Shi Y., Inoue H., Wu J.C., Yamanaka S. (2017). Induced pluripotent stem cell technology: a decade of progress. Nat. Rev. Drug Discov..

[17] Murphy P., Kabir M., Srivastava T., Mason M.E., Dewi C.U., Lim S., Yang A., Djordjevic D., Killingsworth M.C., Ho J.W.K. (2018). Light-focusing human micro-lenses generated from pluripotent stem cells model lens development and drug-induced cataract in vitro. Development.

[18] McColley S.A. (2016). A safety evaluation of ivacaftor for the treatment of cystic fibrosis. Expert Opin. Drug Saf..

[19] Dryden C., Wilkinson J., Young D., Brooker R. (2016). The impact of 12 months treatment with ivacaftor on Scottish paediatric patients with cystic fibrosis with the G551D mutation: A review. Arch. Dis. Child..

[20] Pesudovs K., Elliott D.B. (2003). Refractive error changes in cortical, nuclear, and posterior subcapsular cataracts. Sci. Rep..

[21] Ajenjo M., Domene M., Martínez C. (2015). Refractive changes in nuclear, cortical and posterior subcapsular cataracts. Effect of the type and grade. J. Optom..

[22] Bahrami M., Hoshino M., Pierscionek B., Yagi N., Regini J., Uesugi K. (2015). Refractive index degeneration in older lenses: A potential functional correlate to structural changes that underlie cataract formation. Exp. Eye Res..

[23] Thompson J., Lakhani N. (2015). Cataracts. Prim. Care: Clin. Off. Pr..

[24] Beebe D.C., Holekamp N.M., Shui Y.B. (2010). Oxidative Damage and the Prevention of Age-Related Cataracts. Ophthalmic Res..

[25] Kaimbo Wa Kaimbo D. (2013). Cataracts: Epidemiology, Morphology, Types and Risk Factors.

[26] Brown N.P., Harris M.L., Shun-Shin G.A., Vrensen G.F., Willekens B., Bron A.J. (1993). Is Cortical Spoke Cataract Due to Lens Fibre Breaks? The Relationship Between Fibre Folds Fibre Breaks, Waterclefts and Spoke Cataract. Eye.

[27] Lovicu F.J., Schulz M.W., Hales A.M., Vincent L.N., Overbeek P.A., Chamberlain C.G., McAvoy J.W. (2002). TGFβ induces morphological and molecular changes similar to human anterior subcapsular cataract. Br. J. Ophthalmol..

[28] James E.R. (2007). The Etiology of Steroid Cataract. J. Ocul. Pharmacol. Ther..

[29] Lasa M.S., Podgor M.J., Datiles M.B., Caruso R.C., Magno B.V. (1993). Glare sensitivity in early cataracts. Br. J. Ophthalmol..

[30] Wormstone I.M., Wang L., Liu C. (2009). Posterior capsule opacification. Exp. Eye Res..

[31] Polack S. (2008). Restoring sight How cataract surgery improves the lives of older adults. Community Eye Health..

[32] Watkinson S., Seewoodhary M. (2015). Cataract management: effect on patients’ quality of life. Nurs. Stand..

[33] Rein D.B., Zhang P., Wirth K.E., Lee P.P., Hoerger T.J., McCall N., Klein R., Tielsch J.M., Vijan S., Saaddine J. (2006). The Economic Burden of Major Adult Visual Disorders in the United States. Arch. Ophthalmol..

[34] Dyrda L. Medicare payment for 10 top ASC procedures by case volume. http://https://www.beckersasc.com/asc-coding-billing-and-collections/medicare-payment-for-10-top-asc-procedures-by-case-volume.html.

[35] Cleary G., Spalton D.J., Koch D.D. (2007). Effect of square-edged intraocular lenses on neodymium:YAG laser capsulotomy rates in the United States. J. Cataract. Refract. Surg..

[36] Flaxman S.R., A Bourne R.R., Jonas J.B., Kempen J.H., A Stevens G., Tahhan N., Wong T.Y., Taylor H.R., Bourne R., Ackland P. (2017). Global causes of blindness and distance vision impairment 1990–2020: a systematic review and meta-analysis. Lancet Glob. Heal..

[37] Mahmud I., Kelley T., Stowell C., Haripriya A., Boman A., Kössler I., Morlet N., Pershing S., Pesudovs K., Goh P.P. (2015). A Proposed Minimum Standard Set of Outcome Measures for Cataract Surgery. JAMA Ophthalmol..

[38] Thanigasalam T., Reddy S.C., Zaki R.A. (2015). Factors Associated with Complications and Postoperative Visual Outcomes of Cataract Surgery; a Study of 1,632 Cases. J. Ophthalmic Vis. Res..

[39] Jaffe N. (1986). The bowman lecture: The conquest of cataract: A global challenge. Surv. Ophthalmol..

[40] Truscott R.J.W., Uversky V.N., Fink A. (2007). Eye lens proteins and cataracts. Protein Misfolding, Aggregation, and Conformational Diseases, Part B: Molecular Mechanisms of Conformational Diseases.

[41] Gilliland K.O., Freel C.D., Johnsen S., Fowler W.C., Costello M.J. (2004). Distribution, spherical structure and predicted Mie scattering of multilamellar bodies in human age-related nuclear cataracts. Exp. Eye Res..

[42] Chang J.R., Koo E., Agrón E., Hallak J., Clemons T., Azar D., Sperduto R.D., Ferris F.L., Chew E.Y., Group A.-R. (2011). Risk Factors Associated with Incident Cataracts and Cataract Surgery in the Age-Related Eye Disease Study (AREDS) AREDS Report Number 32. Ophthalmology.

[43] Banh A., Vijayan M.M., Sivak J.G. (2003). Hsp70 in bovine lenses during temperature stress. Mol. Vis..

[44] Dzialoszynski T.M., Milne K., Trevithick J., Noble E. (2016). Heat shock protein concentration and clarity of porcine lenses incubated at elevated temperatures. Mol. Vis..

[45] Barnard S.G.R., McCarron R., Moquet J., Quinlan R., Ainsbury E. (2019). Inverse dose-rate effect of ionising radiation on residual 53BP1 foci in the eye lens. Sci. Rep..

[46] Uwineza A., Kalligeraki A.A., Hamada N., Jarrin M., Quinlan R.A. (2019). Cataractogenic load - A concept to study the contribution of ionizing radiation to accelerated aging in the eye lens. Mutat. Res. Mol. Mech. Mutagen..

[47] Muranov K.O., Poliansky N.B., Kurova V.C., Riabokon A.M., Sheremet N.L., Fedorov A.A., Bannik K.I., Abrosimova A.N., Ostrovsky M.A. (2010). Comparative study on aging, UV treatment, and radiation on cataract formation. Biophysics.

[48] Langford-Smith A., Tilakaratna V., Lythgoe P.R., Clark S.J., Bishop P.N., Day A.J. (2016). Age and Smoking Related Changes in Metal Ion Levels in Human Lens: Implications for Cataract Formation. PLoS ONE.

[49] Becker C., Schneider C., Aballea S., Bailey C., Bourne R., Jick S., Meier C. (2018). Cataract in patients with diabetes mellitus-incidence rates in the UK and risk factors. Eye.

[50] Shiels A., Bennett T.M., Hejtmancik J.F. (2010). Cat-Map: Putting cataract on the map. Mol. Vis..

[51] Graw J. (2009). Genetics of crystallins: cataract and beyond. Exp. Eye Res..

[52] Zhao W.-J.J., Yan Y.-B.B. (2017). Increasing susceptibility to oxidative stress by cataract-causing crystallin mutations. Int. J. Biol. Macromol..

[53] Beyer E.C., Ebihara L., Berthoud V.M. (2013). Connexin mutants and cataracts. Front. Pharmacol..

[54] Berthoud V.M., Ngezahayo A. (2017). Focus on lens connexins. BMC Cell Biol..

[55] Truscott R.J.W. (2005). Age-Related nuclear cataract-oxidation is the key. Exp. Eye Res..

[56] Truscott R.J.W. (2011). Human Age-Related Cataract: A Condition with No Appropriate Animal Model. J. Clin. Exp. Ophthalmol..

[57] Robinson M.L. (2006). An essential role for FGF receptor signaling in lens development. Semin. Cell Dev. Biol..

[58] O’Connor M.D., McAvoy J.W., O’Connor M.D. (2007). In Vitro Generation of Functional Lens-Like Structures with Relevance to Age-Related Nuclear Cataract. Investig. Ophtalmol. Vis. Sci..

[59] Gwon A., Tsonis P.A. (2008). The rabbit in cataract/IOL surgery. Animal Models Eye Research.

[60] Zigler J.Z. (1990). Animal models for the study of maturity-onset and hereditary cataract. Exp. Eye Res..

[61] Beebe D.C. (2008). Maintaining Transparency A Review of the Developmental Physiology and Pathophysiology of Two Avascular Tissues. Semin. Cell Dev. Biol..

[62] Walker J., Menko S.A. (2009). Integrins in lens development and disease. Exp. Eye Res..

[63] Lachke S.A., Maas R.L. (2010). Building the developmental oculome: systems biology in vertebrate eye development and disease. Wiley Interdiscip. Rev. Syst. Boil. Med..

[64] Charlton-Perkins M., Brown N.L., Cook T.A. (2011). The lens in focus: a comparison of lens development in Drosophila and vertebrates. Mol. Genet. Genom..

[65] Augusteyn R.C. (2011). Lens growth and protein changes in the eastern grey kangaroo. Mol. Vis..

[66] Lovicu F.J., McAvoy J.W., De Iongh R.U. (2011). Understanding the role of growth factors in embryonic development: insights from the lens. Philos. Trans. R. Soc. B: Boil. Sci..

[67] Hejtmancik J.F., Riazuddin S.A., McGreal R., Liu W., Cvekl A., Shiels A. (2015). Lens Biology and Biochemistry. Prog. Mol. Boil. Transl. Sci..

[68] Audette D.S., Scheiblin D.A., Duncan M.K. (2017). The molecular mechanisms underlying lens fiber elongation. Exp. Eye Res..

[69] Bassnett S., Costello M.J. (2017). The cause and consequence of fiber cell compaction in the vertebrate lens. Exp. Eye Res..

[70] Bracken M.B. (2009). Why Are So Many Epidemiology Associations Inflated or Wrong? Does Poorly Conducted Animal Research Suggest Implausible Hypotheses?. Ann. Epidemiology.

[71] Shanks N., Greek R., Greek J. (2009). Are animal models predictive for humans?. Philos. Ethic- Humanit. Med..

[72] Ioannidis J.P.A. (2012). Extrapolating from animals to humans. Sci. Transl. Med..

[73] Wolf N., Penn P., Pendergrass W., Van Remmen H., Bartke A., Rabinovitch P., Martin G.M. (2005). Age-related cataract progression in five mouse models for anti-oxidant protection or hormonal influence. Exp. Eye Res..

[74] Bree M., Borchman D. (2018). The optical properties of rat, porcine and human lenses in organ culture treated with dexamethasone. Exp. Eye Res..

[75] Aleo M.D., Avery M.J., Beierschmitt W.P., Drupa C.A., Fortner J.H., Kaplan A.H., Naveta K.A., Shepard R.M., Wlash C.M. (2000). The use of explant lens culture to assess cataractogenic potential. Ann. New York Acad. Sci..

[76] Sampath S., McLean L., Buono C., Moulin P., Wolf A., Chibout S.-D., Pognan F., Busch S., Shangari N., Cruz E. (2012). The Use of Rat Lens Explant Cultures to Study the Mechanism of Drug-Induced Cataractogenesis. Toxicol. Sci..

[77] Wormstone I.M., Eldred J.A., Wormstone M. (2016). Experimental models for posterior capsule opacification research. Exp. Eye Res..

[78] Ibaraki N., Chen S.-C., Lin L.-R., Okamoto H., Pipas J.M., Reddy V.N. (1998). Human Lens Epithelial Cell Line. Exp. Eye Res..

[79] Lauf P.K., Di Fulvio M., Srivastava V., Sharma N., Adragna N.C. (2012). KCC2a Expression in a Human Fetal Lens Epithelial Cell Line. Cell. Physiol. Biochem..

[80] Li X., Dubois D.C., Song D., Almon R.R., Jusko W.J., Chen X. (2017). Modeling Combined Immunosuppressive and Anti-inflammatory Effects of Dexamethasone and Naproxen in Rats Predicts the Steroid-Sparing Potential of Naproxen. Drug Metab. Dispos..

[81] Saraiya N.V., A Goldstein D. (2011). Dexamethasone for ocular inflammation. Expert Opin. Pharmacother..

[82] Laurell C.-G. (2002). Effects of dexamethasone, diclofenac, or placebo on the inflammatory response after cataract surgery. Br. J. Ophthalmol..

[83] Zhang G., Liu S., Yang L., Li Y. (2018). The role of Dexamethasone in clinical pharmaceutical treatment for patients with cataract surgery. Exp. Ther. Med..

[84] Çekiç O., Chang S., Tseng J.J., Akar Y., Barile G.R., Schiff W.M. (2005). Cataract Progression After Intravitreal Triamcinolone Injection. Am. J. Ophthalmol..

[85] Jonas J., Degenring R., Vossmerbauemer U., Kamppeter B. (2005). Frequency of cataract surgery after intravitreal injection of high-dosage triamcinolone acetonide. Eur. J. Ophthalmol..

[86] Gewaily D., Muthuswamy K., Greenberg P.B. (2015). Intravitreal steroids versus observation for macular edema secondary to central retinal vein occlusion. Cochrane Database Syst. Rev..

[87] Sharma A., McKeithan W.L., Serrano R., Kitani T., Burridge P.W., Del Álamo J.C., Mercola M., Wu J.C. (2018). Use of human induced pluripotent stem cell–derived cardiomyocytes to assess drug cardiotoxicity. Nat. Protoc..

[88] Shea B., Swinden M.V., Ghogomu E.T., Ortiz Z., Katchamart W., Rader T., Bombardier C., A Wells G., Tugwell P. (2013). Folic acid and folinic acid for reducing side effects in patients receiving methotrexate for rheumatoid arthritis. Cochrane Database Syst. Rev..

